# Plant-Mediated Effects of Beneficial Microbes and a Plant Strengthener against Spider Mites in Tomato

**DOI:** 10.3390/plants12040938

**Published:** 2023-02-18

**Authors:** Konstantinos Samaras, Soultana Mourtiadou, Theodoros Arampatzis, Myrsini Kakagianni, Maria Feka, Felix Wäckers, Kalliope K. Papadopoulou, George D. Broufas, Maria L. Pappas

**Affiliations:** 1 Laboratory of Agricultural Entomology and Zoology, Department of Agricultural Development, Democritus University of Thrace, 68200 Orestiada, Greece; 2 Laboratory of Plant and Environmental Biotechnology, Department of Biochemistry and Biotechnology, University of Thessaly, 41500 Larissa, Greece; 3 Department of Food Science and Nutrition, University of Thessaly, 43100 Karditsa, Greece; 4 R&D Department, Biobest Group N.V., 2260 Westerlo, Belgium; 5 Lancaster Environment Centre, Lancaster University, Lancaster LA1 4YW, UK

**Keywords:** defense elicitor, pest control, soil microbes, spider mites, tomato

## Abstract

The two-spotted spider mite *Tetranychus urticae* is a polyphagous herbivore with a worldwide distribution, and is a serious pest in tomato and other crops. As an alternative to chemical pesticides, biological control with the release of natural enemies such as predatory mites represent an efficient method to control *T. urticae* in many crops, but not in tomato. Other biological control agents, such as beneficial microbes, as well as chemical compounds, which can act as plant defense elicitors that confer plant resistance against pests and pathogens, may prove promising biological solutions for the suppression of spider mite populations in tomato. Here, we assessed this hypothesis by recording the effects of a series of fungal and bacterial strains and the plant strengthener acibenzolar-s-methyl for their plant-mediated effects on *T. urticae* performance in two tomato cultivars. We found significant negative effects on the survival, egg production and spider mite feeding damage on plants inoculated with microbes or treated with the plant strengthener as compared to the control plants. Our results highlight the potential of beneficial microbes and plant strengtheners in spider mite suppression in addition to plant disease control.

## 1. Introduction

Feeding the increasing human population in a sustainable manner represents a major challenge. If left uncontrolled, herbivorous arthropod pests can be highly destructive to crops, causing significant yield losses, often above 30% [1]. Pesticide application remains the most common method of controlling such pests, despite policies that promote the use of non-chemical methods in crop production. This global trend is in part driven by a strong demand for agricultural products with reduced load of chemicals [2,3,4]. Novel strategies, complementary or alternative to the existing ones, are required to control arthropod pests of crops in the most efficient and environmentally friendly manner.

Biological control, i.e., the use of beneficial agents against harmful organisms, together with breeding for resistance, are the most promising alternatives to chemical control in crop production [5]. Nevertheless, breeding for resistance in modern crops is often hindered by the complex genetic nature of the traits involved, the narrow range of effectiveness (limited to only a few pest species) and the demonstrated ability of pests to overcome resistance mechanisms [6,7,8,9]. Hence, biological control is currently the most widely applied alternative method to control various arthropod pests in organic farming and IPM programs.

Among biocontrol agents, selected root-colonizing microbes (bacteria and fungi) have long been recognized for their ability to antagonize soil-borne pathogens, improve plant growth and nutrition, and also stimulate (prime) the plant immune system against future attackers [10]. Defense priming triggered by soil-borne microbes is generally referred to as Induced Systemic Resistance (ISR). Microbe-mediated ISR is associated with enhanced expression of defense-related genes that only becomes evident upon attack [11,12]. Thus, ISR may provide plants with a cost-effective mechanism of protection against aboveground herbivores [13,14]. For example, an endophytic fungal strain (*Fusarium solani* strain K) was shown to enhance tomato resistance against spider mites [15], indicating that selected microbes can also contribute to the control of important agricultural pests such as insects and mites. Hence, soil-borne beneficial microbes are of particular interest as ‘plant vaccination’ agents, capable of enhancing plant resistance to biotic stressors [16]. Yet, to date, we only have limited and scattered data on the effects of soil-borne beneficial microbes in providing protection to economically important crops against herbivores [17] and soil microbes currently marketed by the biocontrol industry are only provided as plant growth regulators and/or biofungicides.

Plant defenses can also be induced by chemical compounds besides beneficial soil microbes [18,19,20]. Plant strengtheners, for example, include synthetic compounds which are commercially available to improve plant vigor and protect plants against pathogens. Considering that plant defenses against pathogens and herbivores can be mediated by the same signaling pathways, plant strengtheners can be elicitors that also induce resistance against herbivores [21,22,23]. Hence, beneficial soil microbes and plant strengtheners can be efficient alternatives to chemical pesticides in integrated pest management.

Mechanisms involved in plant defense induction by microbes or chemical elicitors may mediate both direct and indirect responses against herbivores [14,24,25]. Direct effects in particular can be directly effective against arthropods; for instance, when they exhibit an increased sensitivity to jasmonic acid (JA) [26,27]. In the present study, we assessed the plant-mediated effects of a series of commercial and laboratory fungal and bacterial strains, as well as the plant strengthener acibenzolar-S-methyl (Table 1), against the two-spotted spider mite *Tetranychus urticae* in tomato. Spider mites are mesophyll cell-content feeders and *T. urticae* is a polyphagous pest that infests a high number of crops of different plant families. Since tomato defenses against spider mites are mediated by the phytohormones JA, salicylic acid and ethylene [28,29], we hypothesized that spider mites could be affected by plant responses elicited by the beneficial microbes and the plant strengthener. To the best of our knowledge, the plant-mediated effects of beneficial soil microbes or plant strengtheners on herbivorous mites have been scarcely addressed so far [30,31,32,33].

## 2. Results

### 2.1. Plant-Mediated Effects on Spider Mite Performance

#### 2.1.1. Spider Mite Performance on Tomato Plants cv. Ace 55

**Figure 1:**
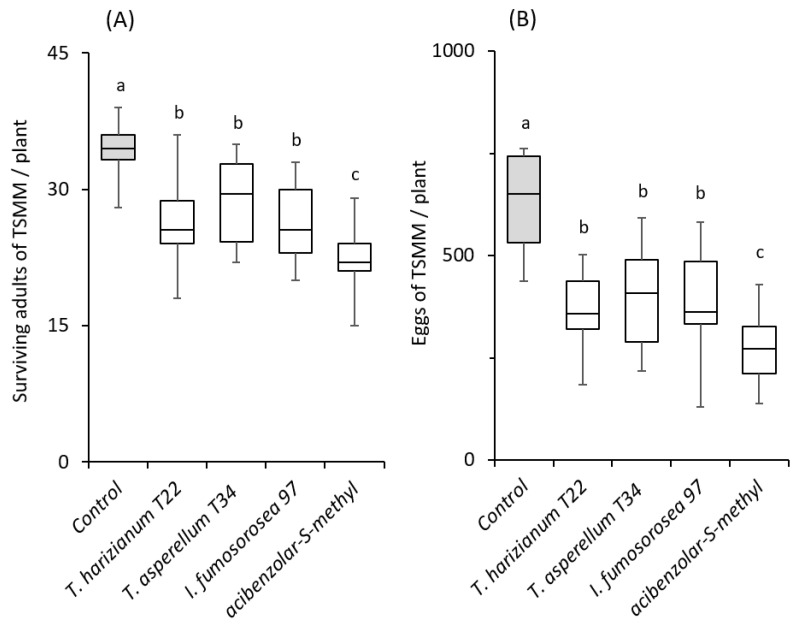
Effects of soil application of beneficial fungi and a plant strengthener on spider mite (*Tetranychus urticae*) performance on tomato cv. Ace 55. Box plots of (**A**) the live adult females and (**B**) spider mite eggs per plant recorded on treated and control plants (n = 18). In each panel, significant differences between treatments are indicated by different letters (Tukey’s HSD, *p* < 0.001).

The number of mites found alive was significantly lower for the plant strengthener treatment, whereas the plants of all other treatments hosted a similar number of spider mites, which was also significantly lower compared to control plants (*F* = 42.75; *df* = 4, 80; *p* < 0.05, Figure 1A).

Furthermore, all fungal strains tested resulted in a significant reduction in spider mite oviposition (*F* = 42.75; *df* = 4, 80; *p* < 0.05, Figure 1B) on tomato plants of the cultivar Ace 55, with females laying approx. 31–50% fewer eggs on treated compared to control plants (*F* = 18.66; *df* = 4, 80; *p* < 0.05, Figure 1B).

Compared to the microbial products, the application of the plant strengthener resulted in an even more pronounced reduction in spider mite oviposition, at approx. 50% of the level observed in the control plants (Figure 1Β).

**Figure 2:**
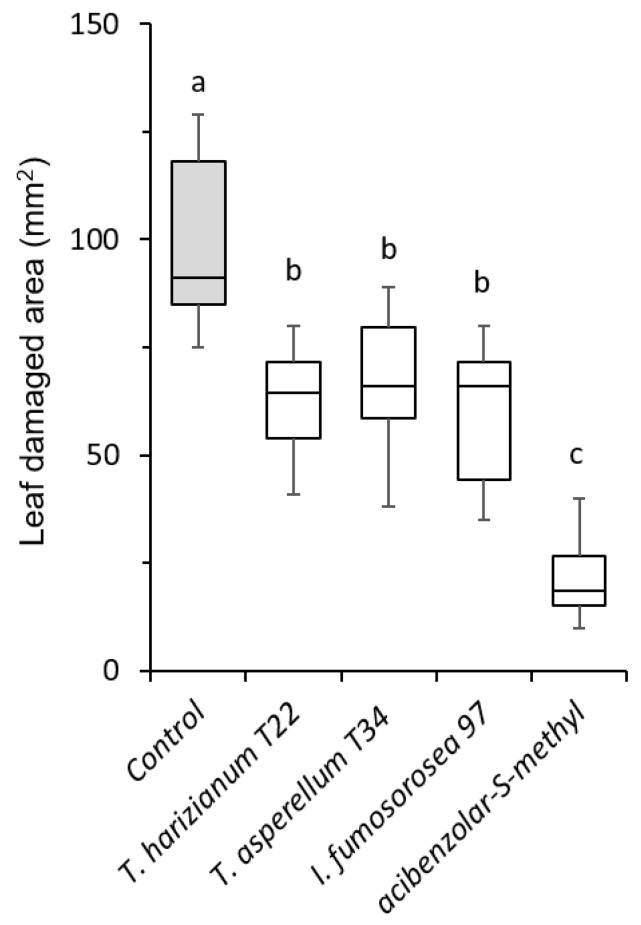
Effects of soil application of beneficial fungi and a plant strengthener on the feeding damage inflicted by spider mites (*Tetranychus urticae*) on tomato cv. Ace 55. Box plots of plant damaged area recorded on treated and control plants (n = 10). In each panel, significant differences between treatments are indicated by different letters (Tukey’s HSD, *p* < 0.001).

Tomato treatment with the different microbial products also resulted in a significant reduction in the damage inflicted by spider mites over the four days of feeding compared to the control plants (Figure 2). Notably, the application of the plant strengthener resulted in the greatest reduction in the feeding damage, both compared to the microbial-treated and the control plants (*F* = 125.02; *df* = 4, 40; *p* < 0.001, Figure 2).

#### 2.1.2. Spider Mite Performance on Tomato Plants cv. Moneymaker

**Figure 3:**
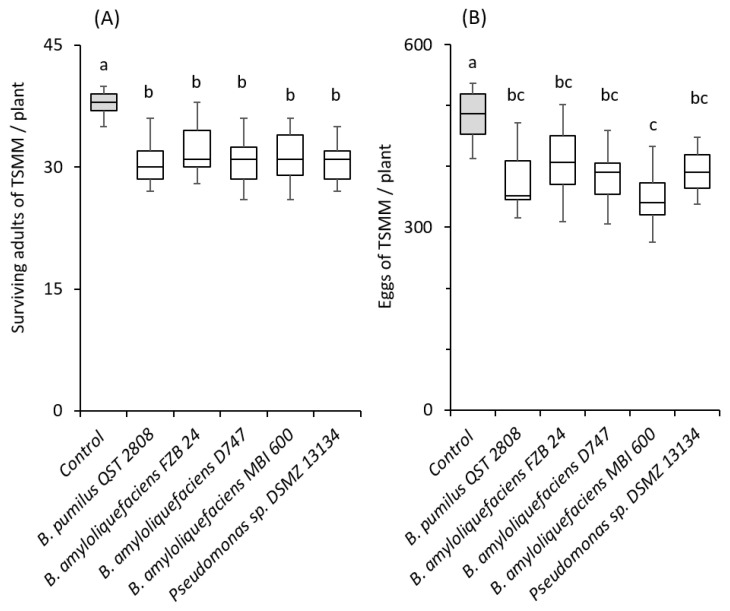
Effects of soil application of beneficial bacteria on spider mite (*Tetranychus urticae*) performance on tomato cv. Moneymaker. Box plots of (**A**) the live adult females and (**B**) spider mite eggs per plant recorded on treated and control plants (n = 15). In each panel, significant differences between treatments are indicated by different letters (Tukey’s HSD, *p* < 0.001).

Bacterial strains tested significantly reduced the number of live spider mites (*F* = 14.27; *df* = 5, 72; *p* < 0.001, Figure 3A), as well as the number of spider mite eggs (*F* = 10.12; *df* = 5, 72; *p* < 0.001, Figure 3B) per plant.

**Figure 4:**
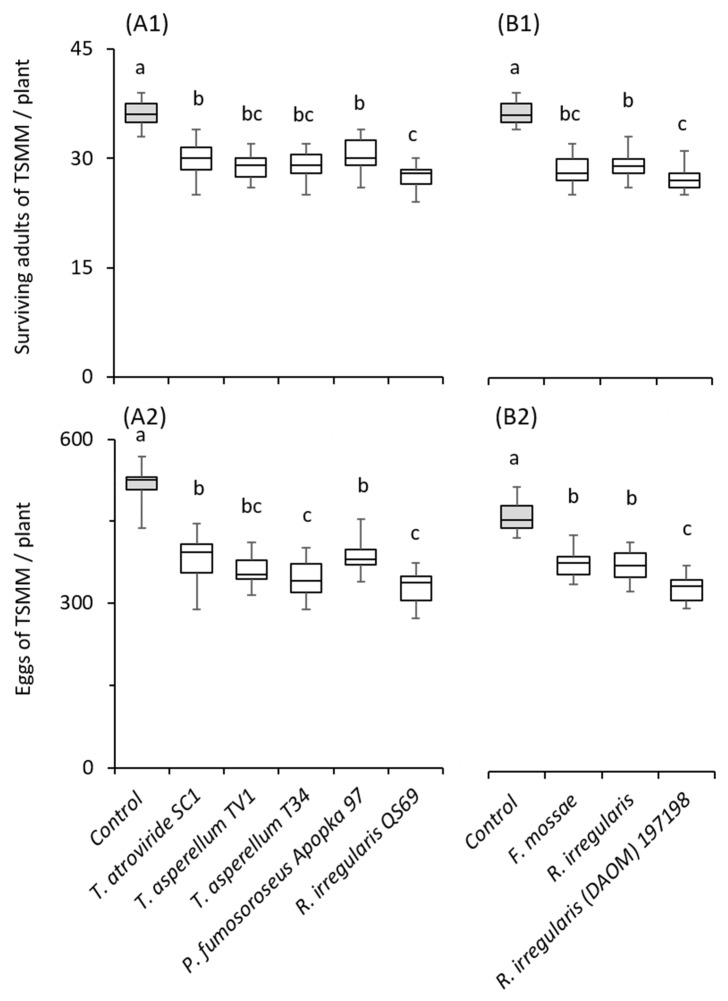
Effects of soil application of beneficial fungi on spider mite (*Tetranychus urticae*) performance on tomato cv. Moneymaker. Box plots of (**A1**,**B1**) the live adult females and (**A2**,**B2**) spider mite eggs per plant recorded on treated and control plants (n = 15). In each panel, significant differences between treatments are indicated by different letters (Tukey’s HSD, *p* < 0.001).

Similarly, all tested fungal strains significantly reduced the number of live spider mites (Figure 4(A1): *F* = 29.76; *df* = 5, 72; *p* < 0.001; Figure 4(B1): *F* = 80.432; *df* = 3, 56; *p* < 0.001), as well as the number of spider mite eggs per plant (Figure 4(A2): *F* = 42.68; *df* = 5, 72; *p* < 0.001; Figure 4(Β2): *F* = 38.05; *df* = 3, 56; *p* < 0.001), with *R. irregularis* QS69 and 197,198 strains resulting in the lowest number of live spider mites and eggs.

Although a direct comparison among the two microbe groups (fungi vs. bacteria) cannot be made, microbes of both groups were shown to result in a similar reduction in spider mites compared with the control (Figure 3 and Figure 4).

### 2.2. Plant Growth Parameters

Overall, no significant effects were recorded in the stem and root weight of tomato plants which were inoculated with the different microbes and infested with spider mites. Stem weight was found to be similar between the different experiments regardless of the bacterial (means ranging from 0.58 to 0.74 g; Microbe (M): *F* = 1.647; *df* = 5, 180; *p* = 0.150; Infestation (I): *F* = 0.255; *df* = 1, 180; *p* = 0.614; M × I: *F* = 0.633; *df* = 5, 180; *p* = 0.675) or the fungal species (means ranging from 0,49 to 0,55 g in group A plants: Microbe (M): *F* = 0.948; *df* = 5, 180; *p* = 0.452; Infestation (I): *F* = 1.089; *df* = 1, 180; *p* = 0.298; M × I: *F* = 0.073; *df* = 5, 180; *p* = 0.996, and from 0,55 to 0,60 g in group B plants: Microbe (M): *F* = 0.946; *df* = 3, 120; *p* = 0.421; Infestation (I): *F* = 0.06; *df* = 1, 120; *p* = 0.937; M × I: *F* = 0.289; *df* = 3, 120; *p* = 0.833).

The same was seen in terms of the weight of the tomato roots, with no significant effects found related to the inoculation of the plants with the different bacterial (means ranging from 0.079 to 0.099 g; Microbe (M): *F* = 1.954; *df* = 5, 180; *p* = 0.88; Infestation (I): *F* = 0.025; *df* = 1, 180; *p* = 0.874; M × I: *F* = 0.176; *df* = 5, 180; *p* = 0.971) or fungal species (means ranging from 0.059 to 0.071 g in group A plants: Microbe (M): *F* = 1.22; *df* = 5, 180; *p* = 0.302; Infestation (I): *F* = 0.551; *df* = 1, 180; *p* = 0.459; M × I: *F* = 0.484; *df* = 5, 180; *p* = 0.788, and from 0,137 to 0,122 g in group B plants: Microbe (M): *F* = 0.601; *df* = 3, 120; *p* = 0.616; Infestation (I): *F* = 0.053; *df* = 1, 120; *p* = 0.819; M × I: *F* = 0.478; *df* = 3, 120; *p* = 0.698)).

## 3. Materials and Methods

### 3.1. Plants

Tomato (*Solanum lycopersicum* L.) plants cv. Ace 55 (Vf) and Moneymaker were used in experiments, as well as in herbivore rearing. Plants were grown from seeds sown in pots (Ø 12 cm) that were filled with sterilized peat (Klasmann-TS2). All plants were maintained in climate chambers (25 ± 2 °C, 16:8 LD, 60–70% RH) and watered every other day. When used in the experiments, plants were 4–5 weeks old.

### 3.2. Herbivores

Spider mites (*Tetranychus urticae*) from laboratory rearing, established with individuals collected from greenhouse tomatoes, were used in the experiments. The mites were reared on detached tomato leaves placed on wet cotton wool in plastic trays at 25 ± 2 °C, 16:8 LD, 60–70% RH. Fresh tomato leaves were added every three days on the trays, which were regularly filled with water as required to maintain leaf vigor. Young female mites (2–4 days old) were used in the experiments. These were obtained by infesting tomato plants with a high number (approx. 300) of female mites that were allowed to lay eggs for 48 h at 25 ± 2 °C, 16:8 LD. The next day, the mites were removed and the plants were maintained at the same conditions until adult mites emerged (after approx. 16 days).

### 3.3. Plant Treatments

#### 3.3.1. Experiments with Tomato Plants cv. Ace 55

We assessed the effects of three commercial fungal products in tomato plants against spider mites and the plant strengthener acibenzolar-S-methyl, a synthetic analogue of salicylic acid (SA) (Table 1). The products were dissolved in water and drenched in sterilized peat in pots where young tomato plants cv. Ace 55 were transplanted 2 days before (10 days from seed sowing). After 3 weeks, the plants were infested on 3 leaflets with spider mites which were reared on ‘Ace 55′ tomato leaflets (15 females per leaflet). Leaflets were selected as described in [28]. Oviposition and survival were recorded 4 days afterwards by removing the infested leaflets and checking them under a stereoscope. During the experiments, the plants were maintained at 25 ± 1 °C, 16:8 LD, 60–70% RH and watered every other day. Two time replicates with nine plants per treatment were used. We used a separate cohort of plants to assess the impact of the treatments on spider mite feeding damage (five plants per treatment, repeated in two independent experiments). In these experiments, plants were infested with 45 spider mite females per plant as described above. Feeding damage was recorded on spider-mite-infested leaflets which were collected and scanned digitally, and damaged leaf area was assessed manually calculated in Photoshop following the steps under ‘Plant Damage Quantification’ as described in [35].

#### 3.3.2. Experiments with Tomato Plants cv. Moneymaker

We assessed the effects of five commercial strains of bacteria and eight strains of fungi, two strains from laboratory and six commercial strains, against spider mites in tomato plants cv. Moneymaker (Table 1). The products (commercial strains) were dissolved in water and drenched in sterilized peat in pots where young tomato plants cv. Moneymaker had been transplanted 2 days before (10 days from seed sowing). Lab fungal strains were routinely cultured on potato dextrose broth (PDB) at 25 °C for 5 days in the dark. Conidial suspensions were prepared and applied as water drench one week after seed sowing as described in Pappas et al. [15]. After 3 weeks, plants were infested with spider mites reared on cv. Moneymaker tomato leaflets as described above. Oviposition and survival were recorded 4 days after inoculation. During the experiments, the plants were maintained at 25 ± 1 °C, 16:8 LD, 60–70% RH and watered every other day. The experiment was conducted three times independently. In each experiment, five plants were used per treatment.

### 3.4. Plant Growth Parameters

Another set of experimental plants cv. Moneymaker was inoculated with beneficial microbes and infested with a standard number of spider mites as described above. Each beneficial microbe was applied in pots with sterilized peat in which tomato plants had been growing. The plants were inoculated with the microbe under study two days after having been transplanted. Ten days after inoculation, the plants were infested with 45 *T. urticae* females per plant. Four days after spider mite introduction, the performance of tomato plants was assessed by recording the dry weight of the above- and belowground plant parts of microbe-inoculated control and herbivore-infested plants.

### 3.5. Statistics

To evaluate the effect of the microbials and the plant strengthener (fixed factor) on the number of spider mite eggs, mite survival and mite damage, a mixed-model ANOVA with replication in time as the random factor was used. In case of significant differences, means were further separated by Tukey’s HSD post hoc test. Similarly, to evaluate the effect of microbial application and infestation by the spider mites (fixed factors) on plant growth parameters (shoot and root dry weight), a mixed-model ANOVA was used with repetition in time as the random factor. Prior to statistical analysis, normality and homogeneity of variances were checked with the Shapiro–Wilk and Levene’s tests, respectively. Significance levels were α = 0.05 for all tests and statistics were performed using SPSS [36].

## 4. Discussion

In the present study, we tested to what extent treating tomato plants with different beneficial microbes or a plant strengthener affects tomato resistance to spider mites. We found that the number of live spider mites was lower on treated compared to control plants, irrespective of the microbial group (bacteria or fungi) or the application of the plant strengthener or the tomato cultivar. In accordance, we recorded a significantly lower egg production and also observed that feeding damage inflicted by spider mites was lower on treated compared to control plants. Finally, plant biomass was not affected by the application of the microbes in herbivore-infested plants compared to the control plants. We argue that these results indicate plant defense induction capabilities in both the tested microbes as well as the plant strengthener, with some variation was recorded between and within the two microbe groups (fungi and bacteria) and between the microbes and the plant strengthener.

Activating the plant’s inherent defense system with the application of beneficial soil microbes or plant strengtheners represents a novel strategy to biologically fend off plant herbivorous pests. Currently, beneficial microbes used against arthropod pests are mainly entomopathogens that typically act on the pest directly. They are known as ‘biopesticides’ in the sense that they are naturally occurring or derived from natural products, and can be formulated and applied on crops in ways similar to conventional pesticides. Among biopesticides, *Bacillus thuringiensis* (*Bt*) is the most widely applied entomopathogenic bacterium against arthropod pests, whereas *Metarhizium*, *Beauveria* and *Isaria* are examples of entomopathogenic fungi. *Pseudomonas*, *Trichoderma* and *Bacillus* (other than *Bt*) are used as biofungicides [24,25,37]. Microbes as biopesticides offer the advantage of lower or no toxicity compared to synthetic pesticides. Nevertheless, their target range can be narrow and even strain-specific. This selectivity of many of the currently available biopesticides means that there is an urgent need for the diversification of the biocontrol toolbox with biocontrol agents that have a wider target-pest range. Beneficial soil microbes and plant strengtheners may offer such an opportunity to impact a broad range of biotic stressors by activating plant defense responses. Among the broad number of currently identified soil bacteria and fungi, a relatively low number of species have been tested for their plant-mediated effects against arthropods, and none of these have reached the biocontrol market in that capacity. The same holds for the plant-mediated effects of plant strengtheners such as acibenzolar-S-methyl, which is commercially available as a fungicide and acts by mimicking the natural systemic acquired resistance of plants against pathogens [38,39,40].

The plant-mediated effects of microbes against spider mites have mainly been studied for entomopathogenic fungi when applied as soil drench or after treating seeds or roots in tomato, bean and strawberry [41,42,43,44,45], and several promising strains of *Metarhizium*, *Beauveria* and *Cordyceps* entomopathogens with plant protection capabilities have been identified. Nevertheless, the plant-mediated effects of other beneficial microbes such as plant-growth-promoting fungi (PGPF) or rhizobacteria (PGPR) on spider mites have been rarely addressed so far. An exception is the study of the beneficial soil endophytic fungus *Fusarium solani* strain K which was shown to negatively affect spider mite performance in tomato via the elicitation of plant defense responses [15], and different PGPR in strawberry [46], as well as the work of Pappas et al. [47], who identified a series of effective beneficial fungi and bacteria against spider mites in pepper. With regard to the effects of arbuscular mycorrhizal fungi (AMF), previous studies have shown variable effects on spider mites. Spider mite performance was shown to be enhanced by the AMF *Glomus mosseae* on bean plants [30,31,32], whereas spider mite performance in *Lotus japonicus* was differentially affected by four different AMF species belonging to different genera depending on the AMF species [48], and negatively affected in citrus plants [49]. It is evident that this important group of plant-interacting organisms need a more thorough evaluation as putative biocontrol agents.

Notably, in our study all fungal strains studied were shown to negatively affect spider mite performance when applied as water drench, while the AMF *Rhizoglomus irregularis* strains were the most promising of all. In addition, the bacteria tested were also shown to negatively affect spider mite performance in tomato. Putative mechanisms involved in the recorded effects could be the production of secondary metabolites, antibiotic effects, feeding deterrents and plant defense induction [16,24,25,41,50,51,52,53], or even the entomopathogenic activity of the microbes colonizing the plant, as has been reported for *C. fumosorosea* [16,54]. Ιn our study, using two different plant cultivars, plant nutritional benefits translated to plant growth were not recorded, while spider mites were negatively affected on microbe-treated plants. In addition, we recorded a difference in the number of live spider mites on plants and non-inoculated control plants, suggesting that recorded differences cannot be attributed to plant responses affecting spider mite reproduction only. Further studies are needed at the molecular and chemical levels to elucidate which of the above mechanisms underlies the reported findings.

Compared to the plant-mediated effects of beneficial microbes, the application of the plant strengthener acibenzolar-S-methyl resulted in more pronounced negative effects on spider mite performance. These effects were reflected in the number of live spider mites and their eggs, as well as at the resulting feeding damage on the acibenzolar-S-methyl-treated plants compared to control plants. Other studies have shown acibenzolar-S-methyl and SA to be involved in induced defense responses against phloem feeders such as aphids in tomato [55,56]. Furthermore, acibenzolar-S-methyl was shown to be effective against mesophyll cell-content feeders such as spider mites when sprayed on tomato and apple trees [38,39,57] or applied in the soil of lima bean plants [58]. Several mechanisms related to the application of acibenzolar-S-methyl have been proposed/demonstrated, ranging from the activation of defense-related enzymes to the expression of pathogenesis-related (PR) genes, as well as the alteration of volatile blend emissions [19,20,39,40]. Studying the molecular and chemical mechanisms involved in tomato–spider mite interactions after acibenzolar-S-methyl application, coupled with behavioral and life-history experiments, will enable us to explain the recorded effects on mite performance.

The plant growth parameters studied in this work were shown not to be affected by the application of the microbes tested. Specifically, dry root and shoot weights of plants were not affected by the application of the microorganisms, irrespective of the spider mite infection. One possible explanation of the absence of effects may be the short duration of the experiments. Studying the effects on plant growth parameters at later stages, i.e., when plants will be inoculated with the microbes under study for longer time periods after transplantation, spanning several weeks or after repeated applications of the microbes, could reveal possible negative or positive effects. On the other hand, the absence of significant effects may be indicative of a trade-off in the plant’s investment in defense responses elicited by soil microbes at the expense of its growth. Specifically with regard to herbivory, the net benefit of microbial application would depend on the trade-off between induced plant defenses versus plant nutritional quality or quantity alteration [14,24,59,60,61]. In the present study, spider mites were adversely impacted on plants treated with the microbes, suggesting the absence of nutritional benefits or that defense induction outcompetes the putative benefits of improved nutrition for the herbivore. Long-term experiments are needed to clarify the plant-growth-promotion effects of the tested microbes versus plant defense induction against aboveground herbivores.

## 5. Conclusions

Collectively, our data support the hypothesis that beneficial soil microbes, as well as the plant strengthener acibenzolar-S-methyl, alter tomato responses to the detriment of the mite population. The putative mechanisms involved should be further explored to assess the extent to which these mechanisms may involve defense induction, priming or plant growth promotion. Our experiments were conducted with tomato plants in pots in sterilized peat under controlled conditions; hence, further experiments in the greenhouse/field could provide additional evidence for the effectiveness of the tested microbes and the plant strengthener in shaping plant–herbivore interactions. The number of spider mite individuals (45 females/plant) used in our experiments to infest plants may resemble the early infestation events when spider mites begin to colonize plants. Accordingly, a previous study suggests an action threshold level of eight mites per leaflet on a second or third recently expanded tomato leaf to avoid yield losses by *T. urticae* [62]. Ultimately, the net benefit of the tested elicitors for the plant and their potential as novel tools in pest control should be confirmed by studying their effects on plant fitness and reproductive output.

## Figures and Tables

**Table 1:** Strains of beneficial microbes and a plant strengthener tested for their plant-mediated effects against spider mites in tomato.

Strain	Origin (Product/Lab)	Dosage (mg/pot)
**Tomato cv: ACE**		
**Fungi**		
*Trichoderma harzianum* T-22	TRIANUM-P^®^ KOPPERT 1 × 10^9^ cfu/g	35
*Trichoderma asperellum* T34	Asperello^®^ T34 Biocontrol^®^, Biobest Group NV 1 × 10^9^ cfu/g	35
*Isaria fumosorosea* Apopka 97	PreFeRal^®^, Biobest Group NV 2 × 10^9^ cfu/g	35
**Plant strengthener**		
Acibenzolar-S-methyl	BION 50 WG Syngenta Hellas	5
**Tomato cv: Moneymaker**		
**Fungi**		
*Isaria fumosorosea* Apopka 97	PreFeRal^®^, Biobest Group NV 2 × 10^9^ cfu/g	0.64
*Trichoderma atroviride* SC1	Vintec^®^, Bi-PA NV/SA 1 × 10^10^ cfu/g	0.09
*Trichoderma asperellum* TV1	Xedavir, Intrachem Hellas 1 × 10^7^ cfu/g	350
*Trichoderma asperellum* T34	Asperello^®^ T34 Biocontrol^TM^, Biobest Group NV 1 × 10^9^ cfu/g	3.50
*Rhizoglomus irregulare* QS69	Advantage, INOQ GmbH 3.6 × 10^4^ propagules/g	10
*Funneliformis mossae*	Lab [34] 2 × 10^5^ cfu/g	10
*Rhizophagus irregularis*	Lab [34] 2 × 10^5^ cfu/g	10
*Rhizophagus irregularis* (DAOM) 197198	DAOM Agronutrition 5 × 10^4^ cfu/mL	10 μL
**Bacteria**		
*Pseudomonas* sp. DSMZ 13134	Proradix^®^, Anthesis 6.6 × 10^10^ cfu/g	0.08
*Bacillus amyloliquefaciens* MBI600	Serifel^®^, BASF Hellas 5.5 × 10^10^ cfu/g	0.32
*Bacillus amyloliquefaciens* FZB24	Taegro ^®^, Syngenta 1 × 10^10^ cfu/g	0.24
*Bacillus pumilus* QST 2808	Sonata ^®^, Bayer 1 × 10^9^ cfu/gr	6.4
*Bacillus amyloliquefaciens* subsp. *plantarum* D747	Amylo-X^®^, K&N Efthymiadis 2 × 10^11^ cfu/g	1.60

## Data Availability

The datasets generated and analyzed during the current study are available from the corresponding author upon reasonable request.
